# Identification of patients with stable chest pain deriving minimal value from coronary computed tomography angiography: An external validation of the PROMISE minimal-risk tool

**DOI:** 10.1016/j.ijcard.2017.09.033

**Published:** 2018-02-01

**Authors:** Philip D. Adamson, Christopher B. Fordyce, David A. McAllister, James E. Udelson, Pamela S. Douglas, David E. Newby

**Affiliations:** a BHF Centre for Cardiovascular Science, University of Edinburgh, Edinburgh, United Kingdom; b Duke Clinical Research Institute, Duke University School of Medicine, Durham, NC, United States; c Division of Cardiology, University of British Columbia, Vancouver, British Columbia, Canada; d Institute of Health and Wellbeing, University of Glasgow, Glasgow, United Kingdom; e The CardioVascular Center, Division of Cardiology, Tufts Medical Center, Boston, MA, United States

**Keywords:** PROMISE, PROspective Multicenter Imaging Study for Evaluation of chest pain, SCOT-HEART, Scottish COmputed Tomography of the HEART, CCTA, coronary computed tomography angiography, CAD, coronary artery disease, CADC, Coronary Artery Disease Consortium, HL, Hosmer-Lemeshow, MICE-PMM, multiple imputation using regression switching with predictive mean matching, Stable angina, Coronary artery disease, Coronary computed tomography angiography

## Abstract

**Background:**

The PROspective Multicenter Imaging Study for Evaluation of chest pain (PROMISE) minimal-risk tool was recently developed to identify patients with suspected stable angina at very low risk of coronary artery disease (CAD) and clinical events. We assessed the external validity of this tool within the context of the Scottish Computed Tomography of the HEART (SCOT-HEART) multicenter randomised controlled trial of patients with suspected stable angina due to coronary disease.

**Methods:**

The minimal-risk tool was applied to 1764 patients with complete imaging and follow-up data. External validity was compared with the guideline-endorsed CAD Consortium (CADC) risk score and determined through tests of model discrimination and calibration.

**Results:**

A total of 531 (30.1%, mean age 52.4 years, female 62.0%) patients were classified as minimal-risk. Compared to the remainder of the validation cohort, this group had lower estimated pre-test probability of coronary disease according to the CADC model (30.0% vs 47.0%, *p* < 0.001). The PROMISE minimal-risk tool improved discrimination compared with the CADC model (c-statistic 0.785 vs 0.730, *p* < 0.001) and was improved further following re-estimation of covariate coefficients (c-statistic 0.805, *p* < 0.001). Model calibration was initially poor (χ^2^ 197.6, Hosmer-Lemeshow [HL] *p* < 0.001), with significant overestimation of probability of minimal risk, but improved significantly following revision of the PROMISE minimal-risk intercept and covariate coefficients (χ^2^ 5.6, HL *p* = 0.692).

**Conclusion and relevance:**

Despite overestimating the probability of minimal-risk, the PROMISE minimal-risk tool outperforms the CADC model with regards to prognostic discrimination in patients with suspected stable angina, and may assist clinicians in decisions regarding non-invasive testing.

**Trial registration:**

ClinicalTrials.gov identifier: NCT01149590

## 1 Introduction

Chest pain is responsible for > 1% of all presentations to family physicians, although stable coronary artery disease (CAD) is the underlying cause in only a minority [1], [2]. Increased community awareness of CAD risk and improvements in primary prevention have led to progressively lower disease prevalence within this patient population and the frequency of abnormal results on ischaemia testing is now < 10% [3]. There is a clear need to refine the assessment of suspected stable angina to optimise the efficient use of diagnostic resources and minimise unnecessary investigations. Recently, investigators from the North American PROspective Multicenter Imaging Study for Evaluation of chest pain (PROMISE) trial developed a risk model to identify individuals at very low risk of CAD [4]. To investigate the generalisability of this risk score, we undertook an external validation in a United Kingdom-based study of computed tomography in the diagnosis of CAD: the Scottish COmputed Tomography of the HEART (SCOT-HEART) trial.

## 2 Methods

The SCOT-HEART study was a prospective multicenter randomised controlled trial investigating the role of coronary computed tomography angiography (CCTA) in patients referred to a specialist clinic with suspected angina due to CAD. The study design [5] and principal findings [6] have previously been reported. Briefly, participants were recruited from 12 cardiology chest pain clinics across Scotland and those randomised to the intervention arm underwent CCTA imaging in addition to routine clinical care. In contrast with PROMISE, there was a higher prevalence of obstructive coronary disease in the SCOT-HEART population (25.4% vs 10.7%) [7]. For the purposes of this analysis, we limited the validation cohort to those individuals randomised to assessment with CCTA who had sufficient data to determine minimal-risk. The mean period of follow-up was 3.3 ± 1.0 years.

Consistent with PROMISE [4], minimal-risk was defined as requiring a coronary calcium score of 0, no CCTA evidence of coronary atherosclerosis, and the absence of any cardiovascular events (including all-cause death, non-fatal myocardial infarction or coronary revascularisation) during follow-up. All variables included in the published model were evaluated in this analysis with the exception of ethnicity because of the disparate ethnic composition of the study populations. The remaining variables include: age, sex, smoking history, diabetes mellitus, dyslipidemia, family history of premature coronary artery disease, hypertension, symptoms related to stress and high-density lipoprotein (HDL) concentration. In cases where HDL-cholesterol concentrations were not available, multiple imputation using regression switching with predictive mean matching (MICE-PMM) was employed [8].

Multivariable binomial logistic regression using the published model coefficients was used to estimate the probability of minimal-risk for each participant and the predicted risk was compared to the observed outcomes for these individuals. Model discrimination was determined from area under the receiver-operator curve (AUC), or c-statistic, and compared to the established CAD Consortium (CADC) risk score, which has recently been demonstrated to outperform the older Diamond-Forrester score [9], [10]. The variables included in the CADC model include age, sex and typicality of presenting symptoms (typical, atypical or non-anginal). *Discrimination* reflects the ability of the model to correctly distinguish between minimal-risk (no plaque and no events) and other-risk individuals (i.e. place all subjects in the correct rank order of risk). *Calibration* describes the agreement between predicted and observed likelihood of minimal risk for an individual. Model calibration was assessed visually by plotting predicted versus observed risk in deciles and quantified with the Hosmer-Lemeshow (HL) statistic. Tests of discrimination and calibration were performed sequentially after step-wise updating of the model intercept (‘recalibration-in-the-large’) and slope to allow for differences in baseline risk between the derivation and validation populations. Finally, these tests were repeated following model revision, retaining the initial covariates but with coefficients re-estimated within the SCOT-HEART cohort. Statistical analysis was performed using R version 3.3.0 (R Foundation for Statistical Computing, Vienna, Austria).

## 3 Results

Within the 2073 participants randomised to the intervention arm, 1778 underwent CCTA scanning. In total, 1764 patients (57.6, SD 9.5 years, female 44%) had complete imaging and outcome data available for analysis of whom 531 (30.1%; 52.4, SD 9.6 years, female 62.0%) fulfilled all criteria for minimal-risk (Supplementary Table 1). High-density lipoprotein cholesterol (HDL-C) concentrations were unavailable in 506 patients (28.7%) and values were imputed.

In comparison with the remainder of the cohort, patients with minimal-risk were less likely to have symptoms of typical angina (24.1% vs 42.6%, *p* < 0.001) and had lower pre-test probability of obstructive CAD as determined from the CADC risk score (30.0% vs 47.0%, *p* < 0.001).

Table 1 reports the observed probability of minimal risk, findings on CCTA, revascularisation and observed probability of all-cause death or non-fatal myocardial infarction within the SCOT-HEART population grouped by decile of predicted probability of no risk.

**Table 1:** Test results and event rates by probability of minimal risk.

Predicted probability of no risk	N	Observed probability of no risk, SD	Findings on CCTA				Revascularisation	Death or MI
			Normal	Mild disease	Moderate disease	Obstructive disease		
0.0–0.1	2 (0.1)	0.00 (0.00)	0 (0.0)	0 (0.0)	0 (0.0)	2 (100.0)	1 (50.0)	0 (0.0)
0.1–0.2	168 (9.5)	0.02 (0.15)	7 (4.2)	27 (16.1)	41 (24.4)	93 (55.4)	43 (25.6)	17 (10.1)
0.2–0.3	313 (17.7)	0.12 (0.33)	54 (17.3)	72 (23.0)	63 (20.1)	124 (39.6)	72 (23.0)	10 (3.2)
0.3–0.4	322 (18.3)	0.18 (0.38)	82 (25.5)	79 (24.5)	55 (17.1)	106 (32.9)	65 (20.2)	10 (3.1)
0.4–0.5	278 (15.8)	0.22 (0.41)	82 (29.5)	68 (24.5)	58 (20.9)	70 (25.2)	41 (14.7)	8 (2.9)
0.5–0.6	250 (14.2)	0.40 (0.49)	115 (46.0)	63 (25.2)	41 (16.4)	31 (12.4)	17 (6.8)	6 (2.4)
0.6–0.7	221 (12.5)	0.56 (0.50)	138 (62.4)	37 (16.7)	27 (12.2)	19 (8.6)	10 (4.5)	3 (1.4)
0.7–0.8	137 (7.8)	0.66 (0.47)	103 (75.2)	15 (10.9)	15 (10.9)	4 (2.9)	1 (0.7)	2 (1.5)
0.8–0.9	60 (3.4)	0.75 (0.44)	50 (83.3)	7 (11.7)	2 (3.3)	1 (1.7)	1 (1.7)	0 (0.0)
0.9–1.0	13 (0.7)	1.00 (0.00)	13 (100.0)	0 (0.0)	0 (0.0)	0 (0.0)	0 (0.0)	0 (0.0)

Data is presented as number (percentage) of patients unless otherwise stated.

SD, standard deviation; CCTA, coronary computed tomography angiography; MI, myocardial infarction.

Model discrimination (Supplementary Fig. 1) was greater using the PROMISE minimal-risk score compared with the CADC model (c-statistic 0.785, 95% confidence interval [95% CI] 0.762–0.808, vs 0.730, 95% CI 0.706–0.755; *p* < 0.001). Discrimination was unaffected by re-calibration of model intercept or slope but the c-statistic improved further following revision of the model coefficients (c-statistic 0.805, 95% CI 0.784–0.827; *p* < 0.001).

**Fig. 1:**
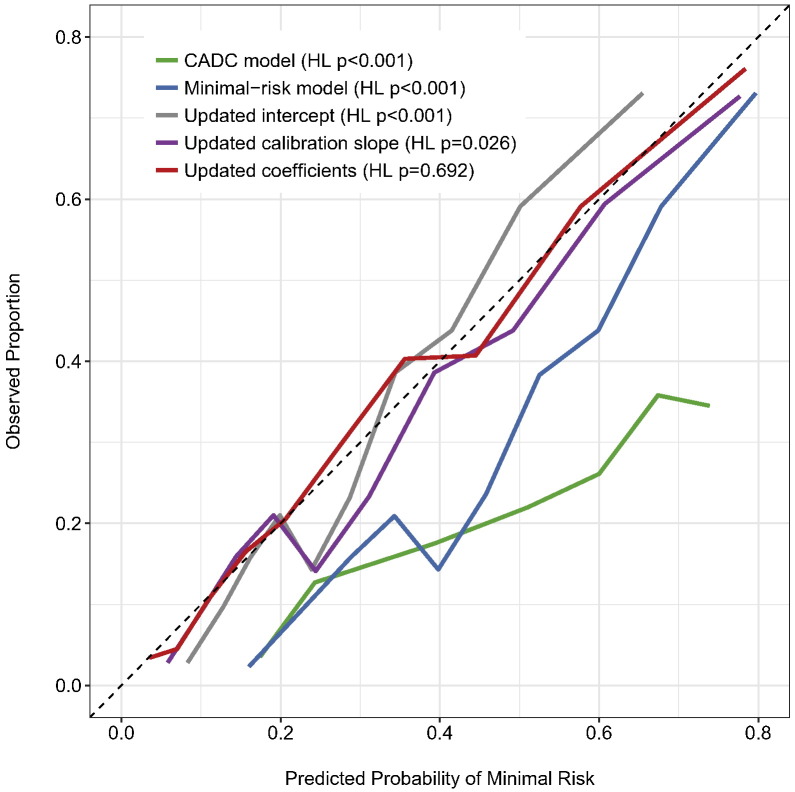
Model calibration. Plot demonstrates poor calibration of predicted probability vs observed proportion of minimal risk using initial model coefficients and intercept (blue) in addition to the established Coronary Artery Disease Consortium (CADC) model (green). Calibration remains poor despite updating the model intercept (grey) and slope (purple). Good model calibration (HL, Hosmer-Lemeshow) is demonstrated once the model coefficients are re-estimated within the validation cohort (red). The dashed line represents perfect calibration [22].

Goodness-of-fit was not demonstrated for either established CADC model (χ^2^ 1010.1, HL *p* < 0.001) or the PROMISE minimal-risk risk tool (χ^2^ 197.6, HL *p* < 0.001). Goodness-of-fit for the PROMISE minimal-risk risk tool remained suboptimal following recalibration of the model intercept (χ^2^ 32.2, HL *p* < 0.001) and calibration slope (χ^2^ 23.7, HL *p* = 0.026) but was improved by the further addition of re-estimated model coefficients (χ^2^ 5.6, HL *p* = 0.692; Supplementary Table 2) resulting in good calibration (Fig. 1).

## 4 Discussion

When applied to the SCOT-HEART population, the PROMISE minimal-risk tool improved model discrimination for excluding CAD when compared with the established CADC model. Indeed, the present c-statistic is greater than that reported in the original PROMISE model derivation (0.785 versus 0.725) [4]. The improved discrimination of the PROMISE score compared with the CADC score likely reflects the different intended purpose of these scores. The CADC model was derived with the objective of accurately estimating the pre-test probability of obstructive coronary artery disease whilst the PROMISE model adopts a broader approach of excluding the presence of any coronary atherosclerosis or any clinical events throughout follow-up.

Model calibration – i.e. the ability to estimate accurately an individuals' absolute risk – was poor when applied in the SCOT-HEART trial population. Such a finding is common when assessing model performance in clinically divergent settings [11], [12], [13], [14] and causes may be patient-related (including differences in case-mix, event rates, or predictor definitions), or model-related, such as over (or under-) fitting of coefficients or omission of important predictive variables.

Poor goodness-of-fit does not necessarily signify lack of model value, and the robust discrimination demonstrated in this report suggests appropriate and informative covariate selection. Therefore, we adopted the recommended stepwise approach to model updating [11] that successfully improved model calibration within the SCOT-HEART population. It is plausible, that the PROMISE risk model remains correct in its initial form for application within the North American context, whilst the updated coefficients may provide more accurate risk estimates for European populations. Regardless, to achieve accurate predictions of absolute risk, it is likely that such population-specific recalibration would be desirable for each setting in which the model was to be applied. However, even in the absence of this recalibration, our findings have important implications for determining appropriate diagnostic pathways and highlight that clinicians need to be aware of the imprecision of the estimates determined from the minimal-risk score. In recognition of this, the current guidelines for prevention of atherosclerotic cardiovascular disease already recommend incorporating explanation of the uncertainty of prognostic models in the clinician-patient risk discussion [15].

In order to understand the reasons for risk estimate imprecision we need to consider carefully the differences in patient populations between the PROMISE and SCOT-HEART trials. Such differences have previously been reported [16], but specific areas of relevance to the current findings warrant mention. First, the improvement in discrimination seen with the SCOT-HEART population is likely to reflect the greater breadth of baseline risk which gives rise to a broader spread of the linear predictors within the validation cohort [17]. Relatedly, there is an apparent difference in the proportion of the trial populations fulfilling the criteria for minimal-risk between the studies, with slightly more low-risk patients identified in the SCOT-HEART cohort. This ‘miscalibration-in-the-large’ is a frequent challenge but can be straightforwardly addressed, if the average patient risk is known within the external clinical setting in which the model is being applied, by adjustment of the model intercept. Furthermore, although the exact nature of the effects of this difference in case-mix or ‘spectrum bias’ is difficult to quantify, some insight can be gained from examination of the change in specific variable coefficients. With one exception, all the coefficients increased in magnitude when re-estimated. Those covariates where the increase was most substantial, for example female sex, appear to be more powerful predictors of minimal-risk in the SCOT-HEART population than was identified in PROMISE.

Ultimately, the clinical value of any prognostic score relies on a previously defined and broadly accepted threshold of risk that suitably assists clinicians in identifying those patients who can have further testing safely deferred. In this regard, the accuracy of absolute predicted risk may be of secondary importance to how reliably a model categorizes or reclassifies an individual into appropriate diagnostic pathways. In the absence of updated clinical guidelines incorporating the minimal-risk tool, it is unclear where this threshold should be set. However, it is reassuring to note that the probability of obstructive coronary artery disease remains < 10% in the highest four deciles of predicted probability of minimal risk, whilst the corresponding risk of death or myocardial infarction remains below 2% in these groups. International guidelines have previously adopted low-risk thresholds in this range, below which further investigation is unhelpful [18], [19]. Should such an approach be continued using the PROMISE model, it would identify 1 in 4 patients who could safely avoid further testing, enabling potentially important reductions in diagnostic resource use.

It should be noted that within the United Kingdom, the National Institute for Health and Care Excellence (NICE) has recently updated guidance for the diagnosis of suspected stable angina [20]. This guideline has endorsed a shift to a symptom-focused approach to chest pain assessment, moving away from the broader cardiovascular risk-based strategy previously recommended [19]. Such an approach appears to more appropriately target the use of non-invasive testing and improve clinical outcomes [21]. Whether the cardiovascular risk assessment provided by the PROMISE risk tool can offer advantages to the NICE approach remains unclear and would need to be prospectively determined.

This study has some limitations. Failure to undergo CCTA as randomised, non-diagnostic images and the absence of coronary calcium scans, precluded the determination of minimal-risk in 309 (14.9%) patients. HDL-C concentrations were not available in a fifth of patients although the MICE-PMM technique for imputation achieves minimally biased estimates and satisfactory model performance with up to 50% missingness [8]. Finally, we excluded unstable angina not leading to revascularisation as a minimal risk exclusion criterion since unlike PROMISE, unstable angina events were not adjudicated in SCOT-HEART. However, with the widespread use of high-sensitivity troponin in Scotland, unstable angina accounts for < 5% of all acute coronary syndromes.

## 5 Conclusion

When assessing patients with suspected stable angina, the PROMISE minimal-risk tool outperforms the CADC model and improves discrimination of the pre-test probability of normal coronary arteries and no clinical events. Suboptimal model calibration may overestimate probability of minimal risk in external populations. Nevertheless, the PROMISE minimal-risk tool may assist clinicians in decisions regarding non-invasive testing.

## Author contributions

Drs Adamson and Newby had full access to all the data in the study and take responsibility for the integrity of the data and the accuracy of the data analysis.

*Study concept and design:* Adamson, McAllister, Newby.

*Acquisition, analysis, interpretation of data:* Adamson, Fordyce, McAllister, Udelson, Douglas, Newby.

*Drafting of the manuscript:* Adamson, Newby.

*Critical revision of the manuscript:* All authors.

## Appendix A Supplementary data

Supplementary material Image 1
