# Supplementary material for: Identification of patients with stable chest pain deriving minimal value from coronary computed tomography angiography: An external validation of the PROMISE minimal-risk tool
Source: Int J Cardiol. 2018 Feb 1;252:31–4. doi: 10.1016/j.ijcard.2017.09.033 (PMC5761719; doi:10.1016/j.ijcard.2017.09.033)
Supplement: Supplementary file 1 — Supplementary material [file mmc1.docx]

**Supplementary appendix**

**Identification of Patients with Stable Chest Pain Deriving Minimal Value from Coronary Computed Tomography Angiography**

***An external validation of the PROMISE Minimal-Risk Tool***

Philip D. Adamson*^a^ MD, Christopher B. Fordyce*^b,c^ MD MHS MSc, David McAllister^d^ MD, James E. Udelson^e^ MD, Pamela S. Douglas^b^ MD and David E. Newby^a^ MD PhD DSc

^a^BHF Centre for Cardiovascular Science, University of Edinburgh, Edinburgh, United Kingdom

^b^Duke Clinical Research Institute, Duke University School of Medicine, Durham, North Carolina

^c^Division of Cardiology, University of British Columbia, Vancouver, British Columbia, Canada

^d^Institute of Health and Wellbeing, University of Glasgow, Glasgow, United Kingdom

^e^The CardioVascular Center, Division of Cardiology, Tufts Medical Center, Boston, Massachusetts

*Equal contribution.

**Correspondence to:**

Dr Philip D. Adamson

Room SU 305

BHF Centre for Cardiovascular Science

Chancellor’s Building

University of Edinburgh

49 Little France Cres

Edinburgh

EH16 4SB

UNITED KINGDOM

Email: [philip.adamson@ed.ac.uk](mailto:philip.adamson@ed.ac.uk)


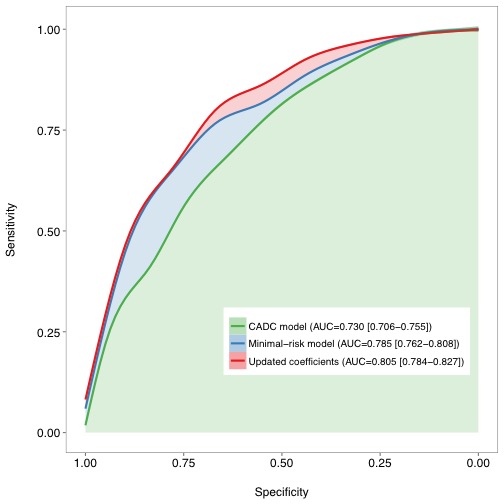


**Supplementary Figure 1: Receiver operating characteristic curve**

The PROspective Multicenter Imaging Study for Evaluation of chest pain (PROMISE) minimal-risk score (blue) demonstrates significantly greater model discrimination compared to the existing Coronary Artery Disease Consortium (CADC) model (green). Discrimination improves further with updated model coefficients (red).

|  | Minimal Risk | Other | P-Value |
| --- | --- | --- | --- |
| n | 531 | 1233 |  |
| Age, y (mean (SD)) | 52.42 (9.59) | 59.79 (8.50) | <0.001 |
| Female | 329 (62.0) | 439 (35.6) | <0.001 |
| Physician estimate of CAD |  |  | <0.001 |
| No | 45 (8.5) | 64 (5.2) |  |
| Unlikely | 334 (62.9) | 453 (36.7) |  |
| Probable | 142 (26.7) | 545 (44.2) |  |
| Yes | 10 (1.9) | 171 (13.9) |  |
| Hypertension | 124 (23.4) | 484 (39.3) | <0.001 |
| Diabetes | 35 (6.6) | 159 (12.9) | <0.001 |
| Dyslipidaemia | 222 (41.8) | 853 (69.2) | <0.001 |
| Family history of premature CAD | 235 (44.3) | 527 (42.7) | 0.591 |
| Peripheral arterial disease | 4 (0.8) | 28 (2.3) | 0.046 |
| Prior stroke/TIA | 12 (2.3) | 64 (5.2) | 0.008 |
| Smoking habit |  |  | <0.001 |
| Never smoked | 295 (55.6) | 550 (44.6) |  |
| Ex-smoker | 136 (25.6) | 453 (36.7) |  |
| Current smoker | 100 (18.8) | 230 (18.7) |  |
| Predicted 10-year CVD risk* | 11.93 (8.45) | 20.52 (11.11) | <0.001 |
| Pre-test probability of obstructive CAD (mean (SD))^ | 27.95 (18.96) | 47.02 (24.53) | <0.001 |
| Chest pain characterisation |  |  | <0.001 |
| Non-anginal | 261 (49.2) | 423 (34.3) |  |
| Atypical angina | 142 (26.7) | 285 (23.1) |  |
| Typical angina | 128 (24.1) | 525 (42.6) |  |
| Symptoms precipitated by stress | 282 (53.1) | 785 (63.7) | <0.001 |
| Symptoms relieved by rest or GTN | 205 (38.6) | 642 (52.1) | <0.001 |

**Supplementary Table 1:** Baseline characteristics

Data is presented as number (percentage) of patients unless otherwise stated.

y, years; CAD, coronary artery disease; TIA, transient ischemic attack; CVD – cardiovascular disease; GTN, glyceryl trinitrate.

BMI, body mass index; CVD, cerebrovascular disease; PVD, peripheral vascular disease; ACE, angiotensin converting enzyme; ARB, angiotensin receptor blocker; ECG, electrocardiogram; CHD, coronary heart disease; PTP, pre-test probability.

*ASSIGN Score (see <http://assign-score.com/)>

^Estimated according to the CAD Consortium risk model

|  |  |  | PROMISE | | | SCOT-HEART | | |
| --- | --- | --- | --- | --- | --- | --- | --- | --- |
|  | Derived coefficients | Updated coefficients | Minimal-risk  N = 829 (26.9%) | Other  N = 2258 (73.1%) | Total  N = 3087 | Minimal-risk  N = 531 (30.1%) | Other  N = 1233 (69.9%) | Total  N = 1764 |
| Age (SD) | -0.083 | -0.095 | 57.8 (7.2) | 61.4 (8.3) | 60.4 (NR) | 52.4 (9.6) | 59.8 (8.5) | 57.57 (9.5) |
| Female | 0.520 | 1.295 | 526 (63.4%) | 1051 (46.5%) | 1577 (51.1) | 329 (62.0%) | 439 (35.6%) | 768 (43.5%) |
| Never smoked | 0.273 | 0.535 | 476 (57.4%) | 1034 (45.8%) | 1510 (48.9%) | 295 (55.6%) | 550 (44.6%) | 845 (47.9%) |
| No diabetes | 0.169 | 0.471 | 694 (83.7%) | 1735 (76.9%) | 2429 (78.7%) | 496 (93.4%) | 1074 (87.1%) | 1570 (89.0%) |
| No dyslipidemia | 0.207 | 0.840 | 324 (39.1%) | 692 (30.6%) | 1016 (32.9%) | 309 (58.2%) | 380 (30.8%) | 689 (39.1%) |
| No family history of premature CAD | 0.171 | 0.276 | 597 (72.0%) | 1486 (65.8%) | 2083 (67.5%) | 296 (55.7%) | 706 (57.3%) | 1002 (56.8%) |
| No hypertension | 0.203 | 0.306 | 351 (42.3%) | 741 (32.8%) | 1092 (35.4%) | 407 (76.6%) | 749 (60.7%) | 1156 (65.5%) |
| Symptoms related to stress | -0.153 | -0.093 | 307 (37.0%) | 1059 (46.9%) | 1366 (44.3%) | 282 (53.1%) | 785 (63.7%) | 1067 (60.5%) |
| HDL, mg/dL (SD) | 0.004 | 0.009 | NR | NR | NR | 54.8 (16.9) | 51.0 (15.2) | 52.1 (15.8) |
| (Intercept) | 3.609 | 2.021 |  |  |  |  |  |  |

**Supplementary Table 2:** Derived and updated minimal-risk model coefficients, and baseline characteristics of these variables within the PROMISE (derivation) and SCOT-HEART (validation) cohorts.

Data is presented as number (percentage) of patients unless otherwise stated.

CAD, coronary artery disease; NR, not reported
